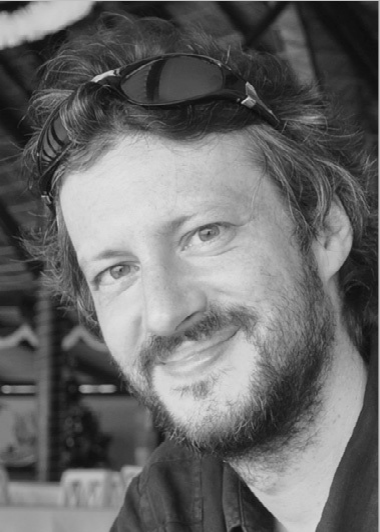# C−H Carboxylation of Aromatic Compounds through CO_2_ Fixation

**DOI:** 10.1002/cssc.201701058

**Published:** 2017-08-16

**Authors:** Junfei Luo, Igor Larrosa

**Affiliations:** ^1^ School of Materials Science and Chemical Engineering Ningbo University Ningbo 315211 P.R. China; ^2^ School of Chemistry University of Manchester Oxford Road Manchester M13 9PL United Kingdom

**Keywords:** base-mediated reactions, carbon dioxide, C−H carboxylation, Lewis-acid-mediated reactions, transition-metal catalysis

## Abstract

Carbon dioxide (CO_2_) represents the most abundant and accessible carbon source on Earth. Thus the ability to transform CO_2_ into valuable commodity chemicals through the construction of C−C bonds is an invaluable strategy. Carboxylic acids and derivatives, the main products obtained by carboxylation of carbon nucleophiles by reaction of CO_2_, have wide application in pharmaceuticals and advanced materials. Among the variety of carboxylation methods currently available, the direct carboxylation of C−H bonds with CO_2_ has attracted much attention owing to advantages from a step‐ and atom‐economical point of view. In particular, the prevalence of (hetero)aromatic carboxylic acids and derivatives among biologically active compounds has led to significant interest in the development of methods for their direct carboxylation from CO_2_. Herein, the latest achievements in the area of direct C−H carboxylation of (hetero)aromatic compounds with CO_2_ will be discussed.

## 1 Introduction

**Scheme 1:**
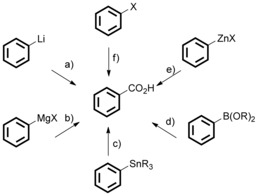
Carboxylation of organometallic compounds. Note that the depicted benzene rings are representative of aryl groups in general.

Carbon dioxide (CO_2_) represents the most abundant carbon source in the Earth′s atmosphere. It is generally considered a green carbon source as it is non‐toxic, low‐cost, and renewable. Furthermore, CO_2_ is proposed to be the major cause of climate change, because of its greenhouse properties and levels of CO_2_ in the atmosphere are steadily increasing. Thus, it is highly attractive to develop methodologies for the transformation of CO_2_ into valuable commodity chemicals.^1^ Particularly, (hetero)aromatic carboxylic acids and derivatives are important motifs among natural products and biologically active compounds.^2^ The catalytic coupling of CO_2_ with energy‐rich substrates, such as epoxides, aziridines, and amines has previously been widely explored for the construction of C−O or C−N bonds.^3^ However, owing to the thermodynamic stability and high oxidation state of CO_2_, its coupling with aromatic compounds its traditionally achieved by the use of highly reactive organolithium or Grignard reagents (Scheme 1 a and b).^4^ These methods suffer drawbacks of low functional‐group compatibility thus limiting their applications. To avoid this issue, many methodologies have been developed over the last two decades for the carboxylation of different organometallic reagents.^5^, ^6^, ^7^ Pioneering work was reported by Nicholas and co‐workers on the insertion of CO_2_ into Sn−C bonds under Pd catalysis although high pressures of CO_2_ were required (Scheme 1 c).^5^ More general methods involved the use of organoboron and organozinc compounds as nucleophiles (Scheme 1 d and e).^6^ Additionally, the groups of Martin, Daugulis, and Tsuji have demonstrated useful methodologies using readily available aryl halides as starting materials for transition‐metal‐catalyzed carboxylation under mild conditions (Scheme 1 f).^7^

C−H functionalization is one of the most encompassing transformations in organic chemistry as it allows efficient and economical access to myriad known and unknown molecules.^8^ In this regard, a more sustainable synthesis of (hetero)aromatic carboxylic acids is the direct insertion of CO_2_ into a C−H bond of an organic substrate. Direct C−H carboxylation methods obviate the need for pre‐functionalized substrates affording, at least in theory, the highest possible step‐ and atom‐economy. Herein, we will review the current state‐of‐the‐art of direct C−H carboxylation of (hetero)aromatic compounds with CO_2_. This Minireview is organized by the type of reagent used in four different sections: 1) base‐mediated carboxylation; 2) Lewis‐acid‐mediated carboxylation; 3) transition‐metal‐catalyzed carboxylation; and 4) enzymatic carboxylation. We have avoided a classification based on the underlying mechanism as often new experiments may reveal a proposed mechanism to be incorrect. However, one can appreciate three main types of carboxylation mechanisms in the reactions discussed herein, depending on the mode of C−H cleavage: 1) an electrophilic aromatic substitution; 2) a C−H deprotonation by base; and 3) a C−H oxidative insertion. The proposed mechanisms for each transformation and their alignment within the three mechanistic classes will be discussed throughout the Minireview.

### 1.1 Base‐mediated carboxylation

The first example of a C−H carboxylation of aromatic compounds was developed by Kolbe and Schmitt during the 1860′s and it is still widely used in industry today. The Kolbe–Schmitt reaction is most notable for the synthesis of Aspirin. It is one of the most important and well‐known carboxylation reactions, providing direct access to salicylic acids through the *ortho* C−H carboxylation of phenoxides with CO_2_.^9^ However, this process generally requires high CO_2_ pressure (20–100 atm; 1 atm=0.101325 MPa) and temperature (130–280 °C) to achieve good conversion. Additionally, the preparation and isolation of completely dry phenoxides from the corresponding phenols is necessary, as the presence of water inhibits the Kolbe–Schmitt carboxylation.^10^ These drawbacks can preclude use in some laboratories. It is generally accepted that in the Kolbe–Schmitt reaction, CO_2_ could be captured by a metal phenolate through weak coordination between the alkali metal and CO_2_.^11^ Water molecules present in the reaction vessel could strongly chelate with the alkali metal phenoxides, consequently preventing the initial addition of CO_2_. The hydrolysis of the alkali metal phenoxides to the phenols may also occur.

**Scheme 2:**
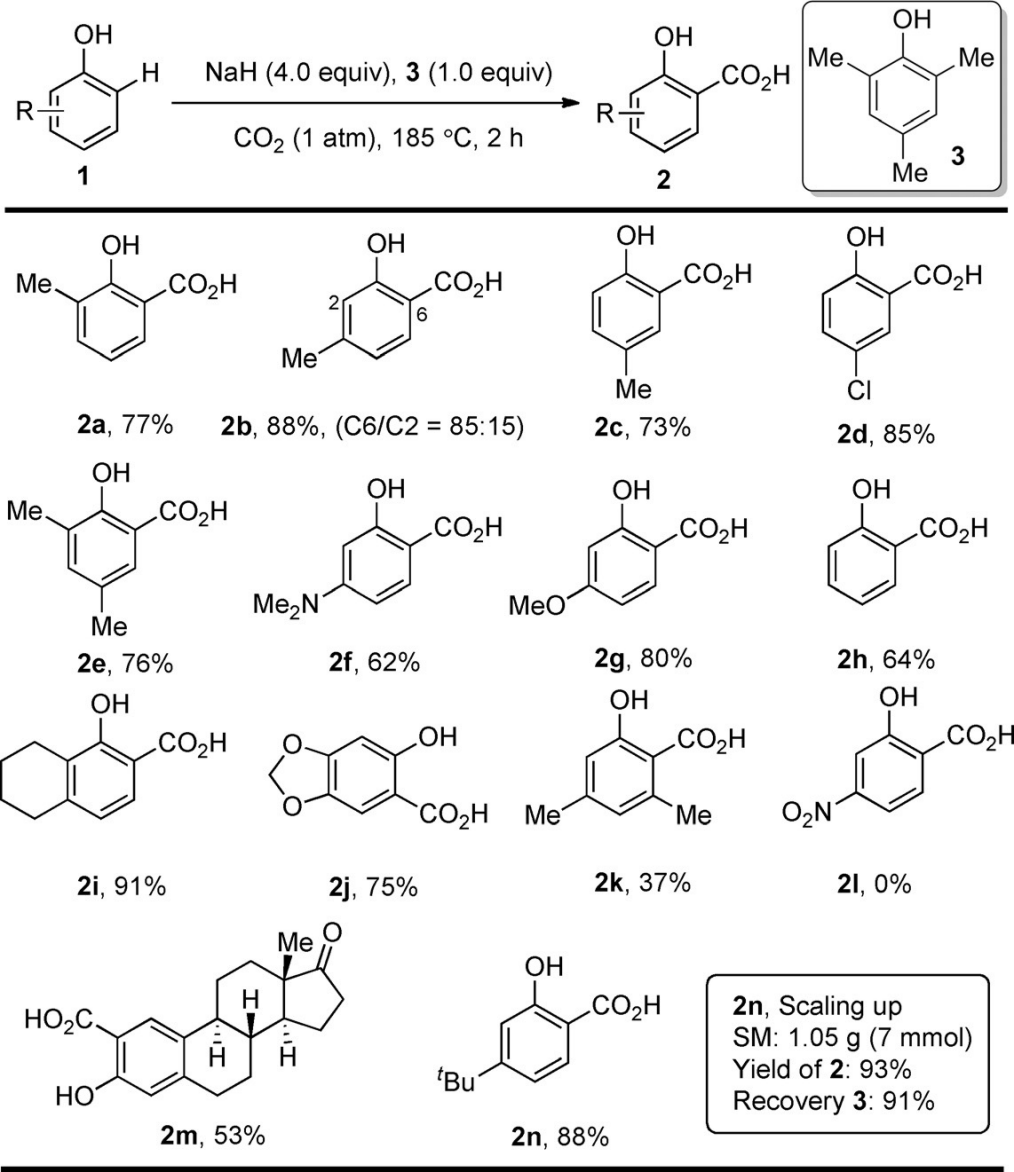
Selected examples of phenol carboxylation. For compound **2 m**: 16 h reaction time.

In 2016, the Larrosa group developed the first protocol for Kolbe–Schmitt‐type carboxylations that occur efficiently under an atmospheric pressure of CO_2_.^12^ The use of NaH as the base avoided the undesired formation of H_2_O, and allowed the reaction to proceed in a one‐pot process without isolation of the phenoxide precursor. More importantly, the authors reported that using 2,4,6‐trimethylphenol (TMP) as a recyclable additive significantly increased the carboxylation rate allowing the process to be carried out under 1 atm of CO_2_. The reaction was compatible with both electron‐donating and halogen substituents, and simple phenol (Scheme 2). However, substrates containing strongly electron‐withdrawing nitro groups were not reactive under these conditions (Scheme 2, **2 l**). It is noteworthy that this procedure is amenable to scaling up and the additive 2,4,6‐trimethylphenol (**3**) can be easily recovered after the reaction (Scheme 2, **2 n**).

**Scheme 3:**
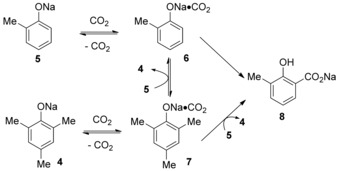
Proposed mechanism for 2,4,6‐trimethylphenol (TMP)‐promoted carboxylation.

The electrophilic aromatic substitution of phenoxide with CO_2_ was the most accepted mechanism for this type of reaction.^11^ It is proposed that sodium 2,4,6‐trimethylphenoxide **4**, generated in situ from **3** and excess NaH, may play a role in aiding CO_2_ fixation, although **4** is inert toward *ortho*‐carboxylation itself (Scheme 3).

**Scheme 4:**
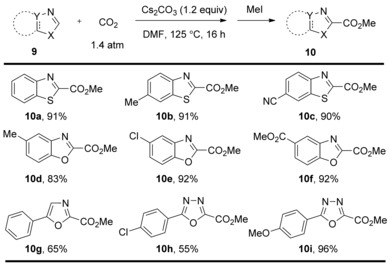
Selected examples of Cs_2_CO_3_‐mediated carboxylation.

Beside the C−H carboxylation of phenol substrates, much attention has been given to the base‐mediated C−H carboxylation of heteroarenes. In this process, heteroarenes bearing sufficiently acidic C−H bonds can undergo carboxylation. In 2010, the research group of Hu developed a methodology for the C−H carboxylation of benzothioazole substrates under 1.4 atm CO_2_.^13^ The group found that the carboxylation proceeds well when using LiO*t*Bu and Cs_2_CO_3_ as bases, whereas K_2_CO_3_, NaOMe, NaOH, and KOH were found to be ineffective. Further investigations revealed that some of the heteroaromatic carboxylic acids are unstable in solution and slowly revert to the heteroarene upon loss of CO_2_. For example, benzothiazole‐2‐carboxylic acid **10 a** shows 20 % decomposition over 5 h. In light of this, the carboxylated compounds were instead isolated as the corresponding methyl esters. The isolation of carboxylation products as the corresponding esters, usually by reaction with an alkyl halide or trimethylsilyldiazomethane (TMSCHN_2_), is a common strategy employed in many of the examples discussed throughout this Minireview. The carboxylation procedure developed by Hu and co‐workers was applicable to benzothiazoles bearing both electron‐withdrawing groups and moderately electron‐donating groups at C6 (Scheme 4, **10 a**–**10 c**). Furthermore, benzoxazole, oxazole, and 1,3,4‐oxadiazole derivatives were also found compatible, leading to the corresponding carboxylic acid derivatives in good yields (Scheme 4, **10 d**–**10 i**).

**Scheme 5:**
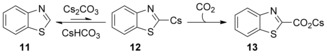
Proposed mechanism for Cs_2_CO_3_‐mediated carboxylation.

The mechanism is somewhat different to the Kolbe–Schmitt reaction; Hu et al. proposed that a Cs_2_CO_3_‐mediated the deprotonation of C−H bond at the C2 position of benzothiazole **11**, followed by reaction with CO_2_ (Scheme 5). The detection of benzothiazole **11** and 2‐benzothiazolecarboxylate **13** by NMR spectroscopy, but not the 2‐benzothiazolyl anion **12**, suggested that the C2‐deprotonation is thermodynamically disfavored.

**Scheme 6:**
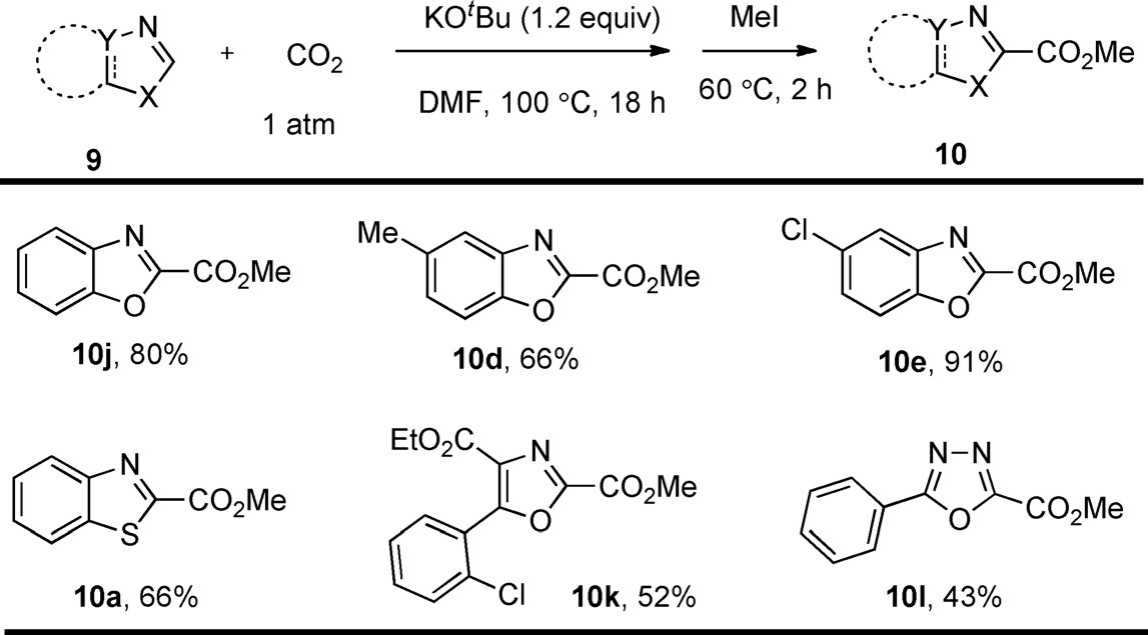
Selected examples of KO*t*Bu‐mediated carboxylation.

More recently, Fenner and Ackermann found that KO*t*Bu enabled the efficient C−H carboxylation of heteroarenes with an ample substrate scope such as benzoxazole, benzothiazole, oxazole, and 1,3,4‐oxadiazole derivatives (Scheme 6).^14^ Compared with the Cs_2_CO_3_‐mediated process (Scheme 4), the use of KO*t*Bu allows for carboxylation at a relatively lower reaction temperature and, more importantly, at only 1 atm CO_2_ pressure. Therefore, this process can be carried out with a simple CO_2_ balloon instead of using specialist high‐pressure equipment. Similar to the Cs_2_CO_3_‐mediated process, the authors suggested the reaction proceeded via initial reversible C−H deprotonation, followed by subsequent CO_2_ insertion.

**Scheme 7:**
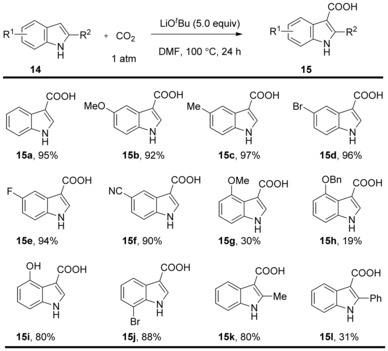
Selected examples for LiO*t*Bu‐mediated carboxylation of indoles.

*tert‐*Butoxide can not only mediate the C−H carboxylation of benzothiazole, benzoxazole, oxazole, and 1,3,4‐oxadiazole derivatives, but also the direct carboxylation of indoles. In 2012, the Kobayashi group found that LiO*t*Bu allowed the formation of indole‐3‐carboxylic acid derivatives under ambient CO_2_ atmosphere.^15^ Interestingly, the authors found the use of a large excess of LiO*t*Bu (5 equiv) was essential to prevent competing product decarboxylation and to improve yields and reproducibility. The process is compatible with both electron‐rich and electron‐poor substituents (Scheme 7) at various positions, albeit substitution at C2 and C4 led to lower yields. The method was subsequently found to be suitable for pyrrole derivatives.^16^

**Scheme 8:**
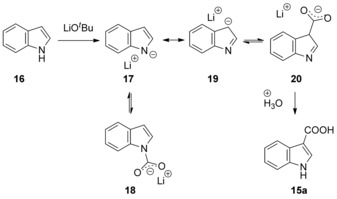
Proposed mechanism for carboxylation of indole.

A tentative mechanism proposed by the authors involves deprotonation at the most acidic N−H proton to anion **17**. This anion can be initially reversibly captured with CO_2_ to form N‐centered carboxylate **18**,^17^ but this compound eventually reacts at the high temperatures of the reaction to form the C3‐carboxylated product **20** (Scheme 8). It is important to note that the free N−H is essential for this carboxylation, as the more nucleophilic N‐methylindole did not undergo base‐mediated carboxylation under the reported conditions. This fact suggested that the reaction may proceed through electrophilic aromatic substitution rather than the deprotonation of the C−H bond at the C3 position of indoles.

The general strategy of base‐mediated carboxylation of (hetero)aromatic compounds requires strong bases such as alkali‐metal hydrides or alkali‐metal *tert*‐butoxides to deprotonate substrates with relatively acidic C−H (or N−H) bonds, such as benzoxazole, oxazole, 1,3,4‐oxadiazole, or indole. However, the direct C−H carboxylation of less acidic (hetero)aromatic compounds with CO_2_ is more challenging. Recently, the research group of Kanan showed that Cs_2_CO_3_ or K_2_CO_3_ molten salts are able to deprotonate poorly acidic C−H bonds (p*K*_a_>40), to afford carbon nucleophiles that can then react with CO_2_ to form carboxylates.^18^ In this regard, furan‐2,5‐dicarboxylic acid (**23**) can be obtained by the carbonate promoted carboxylation of 2‐furoic acid. This is a remarkable transformation as it was previously believed that the deprotonation of 2‐furoic acid could only be achieved with very strong bases such as LDA or *n*BuLi.^19^ Since 2‐furoic acid can be readily made from lignocellulose, this strategy provides a fast and direct route to **23**. The reaction can be conducted under a flow of CO_2_ to give good yield, or under 8 atm of CO_2_ for maximum efficiency (Table 1).

**Table 1:** Carbonate‐promoted carboxylation of 2‐furoic acid. 
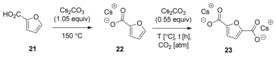

Scale [mmol]	*T* [°C]	CO_2_ [atm]	*t* [h]	**23** [%]
1	260	Flowing	6	57
1	260	Flowing	12	76
1	200	8	5	89
10	195	8	5	78
100	260	1	48	71

**Scheme 9:**
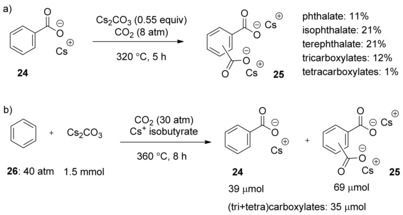
Carbonate‐promoted carboxylation of benzoate (a) and benzene (b).

Encouraged by the results on the carboxylation of 2‐furoic acid, Kanan and co‐workers speculated whether benzoates (substantially weaker acids) would also be suitable substrates. Satisfyingly, when heating the cesium benzoate in the presence of 0.55 equivalents of Cs_2_CO_3_ to 320 °C under 8 atm CO_2_ pressure, a combined yield of 66 % for a mixture of phthalates and tri‐ and tetracarboxylates was obtained (Scheme 9 a). This contrasts with an earlier study that had reported that the carboxylation of cesium benzoate with Cs_2_CO_3_ required much higher CO_2_ pressure (ca. 400 atm) and reaction temperature (380 °C).^20^

The carboxylation of benzene is more challenging than benzoate, owing to the larger entropic penalty and the possible lower solubility of benzene in the molten salt. Kanan and co‐workers reported that carboxylation was possible, in the presence of cesium isobutyrate additive, at 380 °C under 30 atm CO_2_ pressure and 40 atm benzene pressure. It is important to note that the cesium isobutyrate molten component is critical for the success of the reaction, since Cs_2_CO_3_ does not melt at 380 °C in the absence of cesium isobutyrate (Scheme 9 b). The carboxylation of isobutyrate to dimethyl malonate and decomposition of isobutyrate to formate and acetate also took place under these conditions. This work by Kanan and co‐workers shows that the CO_3_^2−^‐promoted carboxylation of benzoate and benzene is possible albeit under extremely forcing conditions.

**Scheme 10:**
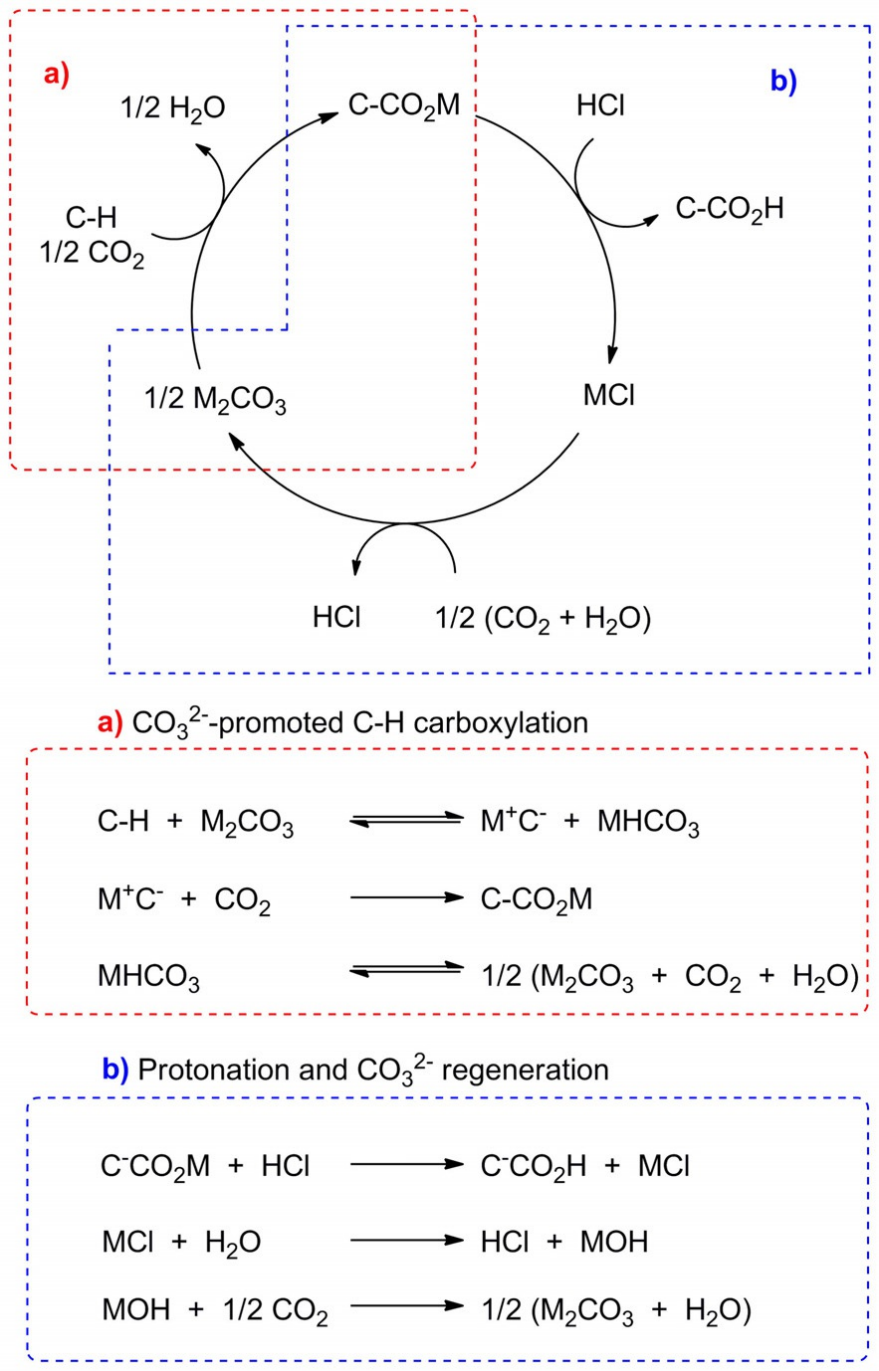
Possible pathways for carbonate‐promoted carboxylation of aromatics.

The same authors envisioned that a M_2_CO_3_ reversibly deprotonates a C−H bond to generate MHCO_3_ and a carbon‐centered nucleophile M^+^C^−^ that could attack CO_2_ to form C−CO_2_M. The decomposition of MHCO_3_ results in a net consumption of 0.5 equiv of M_2_CO_3_ and CO_2_ when per C−CO_2_M produced (Scheme 10 a). The C−CO_2_M could be protonated by the treatment of a strong acid such as HCl to form the carboxylic acid and by‐product MCl. The metal salt MCl could then be processed by electrodialysis to regenerate M_2_CO_3_ and the acid HCl (Scheme 10 b).^21^ In this regard, this whole cycle would effectively transform C−H into C−CO_2_H using only CO_2_ and no other stoichiometric reagents. This protocol would be classified within the C−H deprotonation class by a base‐type pathway.

### 1.2 Lewis‐acid‐mediated carboxylation

**Scheme 11:**
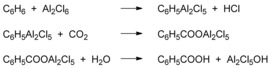
Possible pathways for the carboxylation of benzene promoted by Lewis‐acids.

Friedel and Crafts found that a minor amount of benzoic acid was obtained when CO_2_ was bubbled through a mixture of aluminum chloride and benzene when heated to its boiling point along with the generation of a small amount of hydrogen chloride.^22^ They suggested this process may involve an initial complex between benzene and Al_2_Cl_6_, with subsequent formation of phenyl aluminum dichloride intermediates (PhAl_2_Cl_5_). The organoaluminum species then reacted with CO_2_ to obtain the benzoic acid after aqueous workup (Scheme 11). However, owing to the low electrophilicity of CO_2_ and the side‐reactions attributed to the strong Lewis‐acidity of aluminum‐based compounds, the carboxylic acids are generally obtained in poor yields when using this procedure.^23^ Furthermore, secondary reaction products such as benzophenones and diphenylmethanes are formed in significant amounts.

More recently, Olah and Prakash reported that the use of AlCl_3_/Al can dramatically improve the arene carboxylation yields.^24^ In this procedure, the addition of aluminum metal powder is key, which presumably scavenges the HCl liberated in the process, thus shifting the equilibria toward product formation. Moreover, the AlCl_3_ generated in situ upon reaction of Al with HCl further promotes carboxylation. This direct carboxylation could be carried out at moderate temperatures in good‐to‐excellent yields. The more active substrates such as mesitylene underwent the carboxylation to the corresponding acid with 80 % yield even at 20 °C. Conversely, deactivated aromatics such as benzene halides gave relatively low yields and nitrobenzene substrates were not carboxylated (Table 2).

**Table 2:** AlCl_3_/Al promoted‐carboxylation of benzene derivatives. 
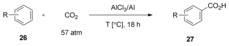

Reactant	*T* [°C]	Main product	Yield^[a]^ [%]	Selectivity [% *o*/*m*/*p*]
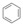		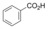		
**26 a**	70	**27 a**	88	–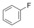		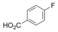		
**26 b**	40	**27 b**	47	1/1/98
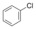		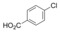		
**26 c**	60	**27 c**	45	3/2/95
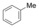		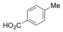		
**26 d**	70	**27 d**	69	7/3/90
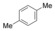		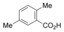		
**26 e**	40	**27 e**	92	–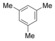		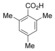		
**26 f**	20	**27 f**	80^[b]^	–

[a] Yields are based on the quantity of AlBr_3_. [b] 68 h reaction time.

**Scheme 12:**
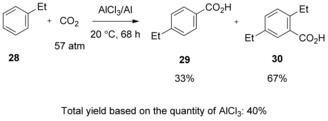
AlCl_3_/Al**‐**promoted carboxylation of alkylbenzenes.

Unfortunately, among alkylbenzenes only methylbenzenes are suited toward selective carboxylation as other homologues tend to disproportionate when treated with anhydrous AlCl_3_/Al, decreasing the yield of the desired carboxylic acids (Scheme 12). It is important to note that, unlike the previous report that led to the formation of a significant amount of diaryl ketone,^25^ this procedure only produced a small amount of those side products.

**Scheme 13:**
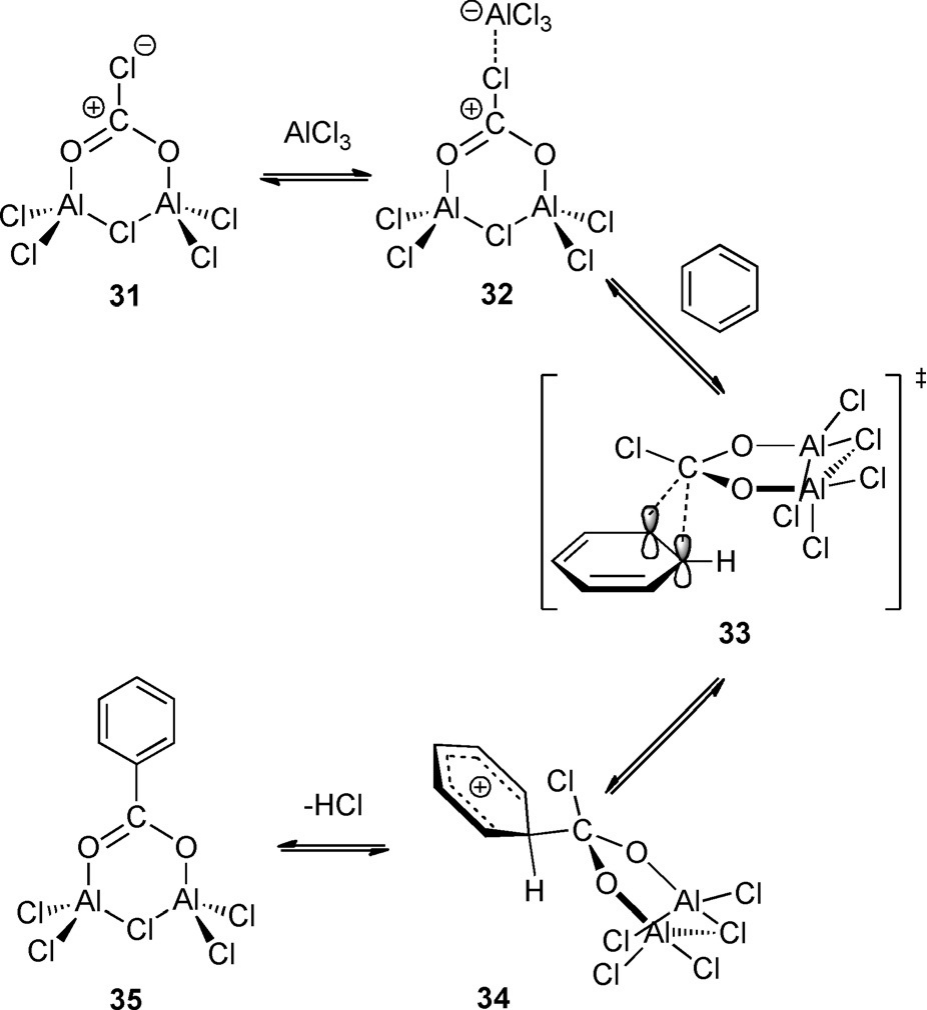
Possible pathways for AlCl_3_/Al**‐**promoted carboxylation of alkylbenzenes.

To investigate the reaction mechanism, density functional theory (DFT) studies on the activation process were carried out. The calculations favor a pathway involving the formation of a CO_2_–AlCl_3_ complex over the previously proposed formation of PhAlCl_2_. On the basis of these calculations, a new mechanistic pathway was proposed. Initial activation of CO_2_ by two molecules of aluminum chloride afford six‐membered ring CO_2_–(AlCl_3_)_2_ complex **31**. In the presence of a further AlCl_3_ molecule, **31** would react with benzene via transition state **33** to give intermediate **34**. Finally, re‐aromatization with loss of HCl affords aluminum carboxylate **35** (Scheme 13). More recently, Munshi and co‐workers found that the carboxylation of toluene proceeded more efficiently by holding AlCl_3_ under pressured CO_2_ (ca. 70 atm) for 1 h prior to the addition of toluene. This manipulation enables the carboxylation to occur with weaker Lewis acids, such as SnCl_4_, MoCl_5_, and TiCl_4_ without appreciable loss of the product yield.^26^ These observations further support Olah′s mechanism of Lewis‐acid activation of CO_2_ rather than the activation of aromatic ring. This mechanism is similar to that of the Kolbe–Schmitt reaction proceeding via an electrophilic aromatic substitution pathway.

**Scheme 14:**
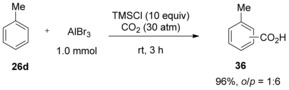
Lewis‐acid‐mediated carboxylation in the presence of TMSCl.

The research group of Hattori, on the other hand, found that the carboxylation of aromatic compounds with CO_2_ can be significantly promoted by the addition of a large excess of chlorotrimethylsilane (TMSCl), giving the corresponding carboxylic acids in good‐to‐excellent yields. It is noteworthy that these reaction conditions are mild, proceeding under room temperature at 30 atm of CO_2_ pressure (Scheme 14).^27^

**Scheme 15:**
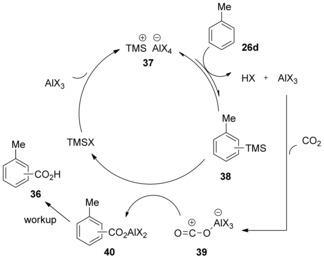
Proposed mechanism for trimethylsilyl choride (TMSCl)‐promoted carboxylation.

The authors suggested that TMSX (X=Cl or Br) could be activated in the presence of the AlX_3_ to form TMSAlX_4_ (**37**). These species would then silylate the aromatic substrate through an electrophilic aromatic substitution, affording arylsilane **38** and recovering AlX_3_. Subsequently, CO_2_‐AlX_3_ complex **39** can react with arylsilane **38** to give aluminum carboxylate **40**, simultaneously regenerating TMSX at the expense of an equimolar amount of AlX_3_ (Scheme 15). This step would be favored by the stabilizing effect of the Si−C bond on the developing positive charge (the β‐effect). The authors propose that the silylation step is an equilibrium process, thus justifying the requirement of a large excess of TMSX reagent to ensure the reaction proceeds in high yields. However, this explanation for the need of a large excess of TMSX seems unlikely if, as proposed, the step forming **37** is irreversible as the quantity of **37** would be determined by the concentration of limiting reagent AlX_3_.

**Scheme 16:**
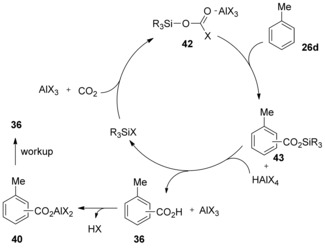
Revised mechanism for R_3_SiX‐promoted carboxylation.

Subsequently, Hattori′s group further extended this work showing that a variety of trialkyl‐ or triaryl‐silyl chlorides efficiently promote the AlBr_3_‐mediated carboxylation of aromatic compounds.^28^ Triphenyl silyl chloride performed best among the silyl chlorides tested and, especially when polycyclic arenes were used, almost quantitative yield was obtained with high regioselectivity. On the basis of the recovery of Ph_3_SiCl at the end of the reaction, the authors suggested that the silyl halides may act as catalysts in the carboxylation, although they are required in excess. In a revision to their previous proposal, the authors suggested that silyl chlorides promote carboxylation by reacting with CO_2_ in cooperation with AlX_3_ to give haloformate‐like active species **42**, which could react with the arene to give a silyl ester **43** and the superacid HAlX_4_. The decomposition of **43** in the presence of HAlX_4_ leads to the carboxylic acid and regenerates the trialkylsilyl chloride R_3_SiX. Finally, the carboxylic acid reacts with AlX_3_ to afford an aluminum carboxylate **40** at the expense of an equimolar amount of AlX_3_ (Scheme 16). This mechanistic proposal would fall within the electrophilic aromatic substitution‐type class.

**Scheme 17:**
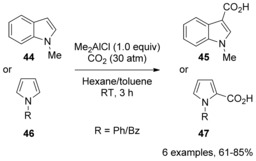
Lewis‐acid**‐**mediated carboxylation of indoles and pyrroles.

1‐Substituted indoles and pyrroles can also be efficiently carboxylated under CO_2_ pressure (ca. 30 atm) by using dialkylaluminum halides (Me_2_AlCl) instead of aluminum trihalides.^29^ This method is applicable to the regioselective carboxylation of 1‐methylindoles, 1‐benzylpyrroles, and 1‐phenylpyrroles to afford the corresponding indole‐3‐carboxylic acids and pyrrole‐2‐carboxylic acids in good yields (Scheme 17). Unprotected simple indole or pyrrole could also undergo carboxylation but low yields resulted.

**Scheme 18:**
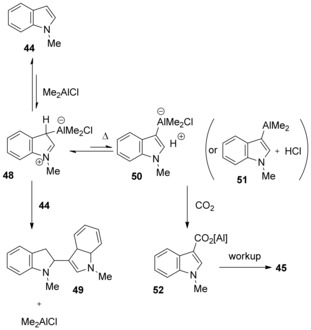
Proposed mechanism for the carboxylation of indoles.

Hattori et al. suggested that zwitterionic species **48** was formed initially through electrophilic addition of Me_2_AlCl to 1‐methylindole, followed by deprotonation to ate complex **50**, and carboxylation (Scheme 18). The equilibrium between **44**, **48**, and **50** is proposed to lie significantly toward the starting materials, the equilibrium being displaced by the carboxylation under high pressure of CO_2_. Notably, HCl may be eliminated from zwitterionic species **48** to form indolylaluminum species **51** instead of ate complex **50**.^30^ Ingleson and co‐workers reported that treatment of 1‐methylindole **44** with AlCl_3_ at 80 °C afforded zwitterionic species similar to adduct **48**, as evidenced by X‐ray analysis.^31^

The same treatment of thiophenes and benzothiophenes with EtAlCl_2_ under pressurized CO_2_ gave the corresponding carboxylic acids in up to 90 % yield.^32^ Also, fused‐ring aromatic compounds, such as naphthalene, anthracene, and phenanthrene, could undergo Lewis‐acid‐mediated carboxylation under CO_2_ pressure (ca. 30 atm), giving 1‐naphthoic acid, 9‐anthracenecarboxylic acid, and 9‐phenanthrenecarboxylic acid, respectively.^33^ However, placing an electron‐withdrawing group on the fused‐ring aromatic compounds completely hindered carboxylation. A plausible mechanism may involve the attack of a Lewis‐acid‐activated CO_2_ molecule on the aromatic ring to form an arenium ion, followed by re‐aromatization to give a carboxylic acid after aqueous workup.

**Scheme 19:**
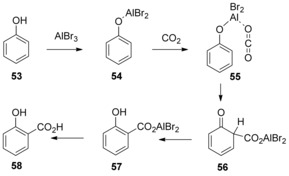
Proposed mechanism for the carboxylation of phenol, mediated by a Lewis acid.

Salicylic acid derivatives have long been synthesized using the Kolbe–Schmitt reaction mediated by a base (see Section 2). However, Iijima and Yamaguchi found that the carboxylation of phenol with CO_2_ (ca. 80 atm) could also take place in the presence of a Lewis acid at moderate temperatures.^34^ Among the Lewis acids investigated, AlBr_3_ was found to be the most efficient, leading to the salicylic acids in approximately 70 % yields. The proposed reaction mechanism involved the formation of phenoxyaluminum dibromide **54** from phenol in the presence of AlBr_3_. The intermediate **54** could react with CO_2_ to produce the CO_2_ complex, which after *ortho*‐carboxylation and re‐aromatization afforded the aluminum salt of salicylate **57**. Simple workup of **57** furnished the salicylic acid **58** (Scheme 19). This proposed mechanism is similar to the Kolbe–Schmitt reaction, proceeding via an electrophilic aromatic substitution.

**Scheme 20:**
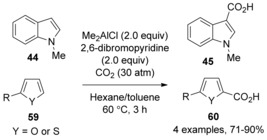
EtAlCl_2_/2,6**‐**dibromopyridine‐promoted carboxylation.

It may be expected that bases would be incompatible with Lewis‐acid‐mediated carboxylations. However, Tanaka, Hattori, and co‐workers developed a method for C−H carboxylation with CO_2_ mediated by a combination of EtAlCl_2_ and 2,6‐dibromopyridine as a weak base.^35^ This method allows indole, thiophene, and furan derivatives to be carboxylated to the corresponding carboxylic acids (Scheme 20). It is important to note that the protocol also enables a variety of α‐arylalkenes and trialkyl‐substituted alkenes to undergo carboxylation with CO_2_ to afford the corresponding α,β‐and/or β,γ‐unsaturated carboxylic acids.

**Scheme 21:**
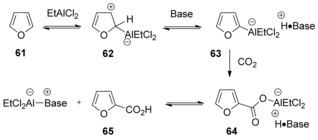
Proposed mechanism for EtAlCl_2_/2,6**‐**dibromopyridine**‐**promoted carboxylation.

The authors suggested that zwitterion **62** could be generated via electrophilic addition of starting material **61** to EtAlCl_2_. Then, 2,6‐dibromopyridine would abstract a proton to afford ate complex **63**. Carboxylation of the ate complex **63** affords the aluminum carboxylate **64**. Owing to the far greater acidity of the conjugate acid of the pyridine base (p*K*_a_≈−2) compared with the carboxylic acid (p*K*_a_≈5), the carboxylic acid **65** could be liberated from the aluminum carboxylate **64** (Scheme 21). Apparently, the use of 2,6‐dibromopyridine base favors the dissociation of the aluminum–pyridine salt owing to its sterically bulky nature and lower basicity.

### 1.3 Transition‐metal‐catalyzed carboxylation

#### 1.3.1 Au‐catalyzed C−H carboxylation

During recent decades, transition‐metal catalysis has set the stage for efficient direct C−C bond‐forming reactions to proceed under exceedingly mild reaction conditions. They have been extended to include site‐selective direct carboxylation of C−H bonds. Our group has shown that the gold complex LAuCl was able to cleave the most electron‐deficient C−H bond of electron deficient (hetero)aromatics to provide the corresponding aryl–Au^I^ species. This allowed for the first general protocol for direct C−H activation of a variety of relatively acidic (hetero)arenes with Au^I^ complexes via a concerted metalation deprotonation.^36^ A variety of ligands (L) such as alkyl and aryl phosphines, phosphites, and N‐heterocyclic carbenes (NHCs) were found to be compatible with this procedure. Simultaneously, the research group of Nolan showed that a monomeric, dicoordinate, linear Au^I^/NHC hydroxide complex was capable of inducing C−H activation on (hetero)arenes.^37^

**Scheme 22:**
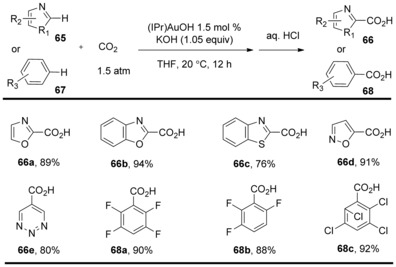
Selected examples of (NHC)AuOH‐catalyzed direct C−H carboxylation.

Carrying on from this work, Nolan and co‐workers reported that the N‐heterocyclic carbene gold complex enabled C−H carboxylation of (hetero)arenes with CO_2_ at ambient temperature in the presence of a stoichiometric amount of KOH base and only 1.5 atm of CO_2_ pressure.^38^ The IPr (1,3‐bis(2,6‐diisopropylphenyl)imidazol‐2‐ylidene) gold complex (IPr)AuOH was found to be the most efficient of all examined, leading to the carboxylated products in good‐to‐high yields. This methodology allowed direct C−H carboxylation of carbocycles and heterocycles including oxazoles, thiazoles, isoxazoles, and some aromatic heterocycles containing multiple heteroatoms (Scheme 22, **66 a**–**e**). The C−H activation is highly regioselective at the most acidic C−H bond. Additionally, this transformation is efficient for polyfluoro‐ and polychloro‐substituted benzene derivatives (Scheme 22, **68 a**–**c**).

**Scheme 23:**
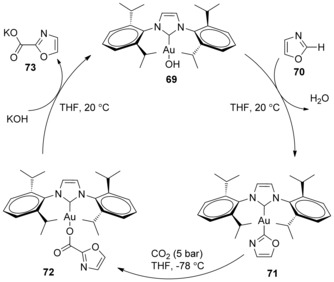
Proposed mechanism for Au‐catalyzed C−H carboxylation.

Stoichiometric reactions were carried out to explore the mechanism of this transformation by the Nolan group. By simply mixing gold complex (IPr)AuOH **69** with oxazole (**70**), 93 % of gold–oxazole species **71** was obtained. The insertion of CO_2_ (5 atm, −78 °C) into the C−Au bond by nucleophilic addition of oxazole to the electrophilic CO_2_ carbon atom produced the carboxylate complex **72** in 86 % yield. Finally, the metathesis of carboxylate complex **72** with KOH regenerated the (IPr)AuOH catalyst and simultaneously released the potassium carboxylate **73** (Scheme 23). This method is related to the Cs_2_CO_3_‐mediated carboxylations, proceeding by C−H deprotonation. Contrarily to this proposal, Ahlquist, Wendt, and co‐workers showed that the reactivity of (NHC)Au^I^Ar complexes with strongly electrophilic substrates such as methyl triflate or methyl iodide to form toluene, biphenyl, and ethane were most likely to occur through an oxidative mechanism. Based on experimental and computational results, Ahlquist, Wendt, et al. argued that the Au−C σ bond of (NHC)Au^I^Ar complexes exhibit poor nucleophilicity and suggested that they would be unlikely to react with CO_2_.^39^

#### 1.3.2 Cu‐catalyzed C−H carboxylation

**Scheme 24:**
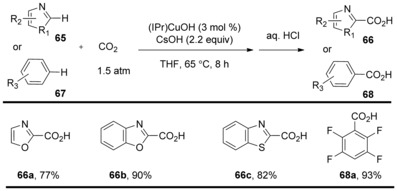
Selected examples of (IPr)CuOH‐catalyzed direct C−H carboxylation.

Nolan′s group later improved upon this methodology by the use of a cheaper copper‐based (IPr)CuOH catalyst instead of a gold complex.^40^ This methodology allows heterocycles including oxazoles, thiazoles, and polyfluorobenzenes to undergo C−H carboxylation with CO_2_ at the most acidic position (Scheme 24). This copper‐based system permits a significant range of N−H carboxylation (p*K*_a_<27.7) such as imidazole, indole, and pyrazole derivatives, which were transformed cleanly and quantitatively to the corresponding methyl esters. Preliminary mechanistic studies suggest a mechanism analogous to that proposed when using (IPr)AuOH catalyst (Scheme 23).

**Scheme 25:**
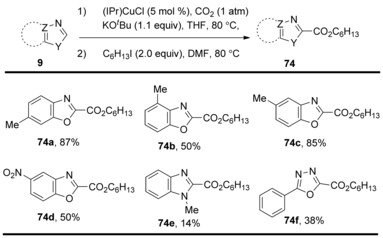
Selected examples of (IPr)CuCl‐catalyzed direct C−H carboxylation.

At the same time, Hou and co‐workers reported a similar C−H carboxylation using (IPr)CuCl catalyst.^41^ Various benzoxazole derivatives bearing both electron‐donating and electron‐withdrawing groups as the aryl unit could be carboxylated in good‐to‐high yields under atmospheric CO_2_ pressure (Scheme 25). More recently, the authors found that 1,2,3‐triazol‐5‐yideneb (*tz*NHC)‐based copper complexes, such as 1,4‐di(2,6‐diisopropylphenyl)‐3‐methyl‐1,2,3‐triazol‐5‐ylidene‐copper chloride (TPr)CuCl could promote the carboxylation reaction somewhat more effectively than the corresponding imidazol‐2‐ylidene copper(I) complex (IPr)CuCl. This allowed benzoxazole and benzothiazole derivatives to undergo C−H carboxylation with CO_2_ to give the corresponding esters in excellent yields after treatment with an alkyl iodide.^42^ The research group of Gooßen developed an easily accessible and relatively stable catalyst, namely (4,7‐diphenyl‐1,10‐phenanthroline)bis‐[tris(4‐fluorophenyl)‐phosphine] copper(I) nitrate for the smooth carboxylation of benzoxazole at standard atmospheric CO_2_ pressure. Furthermore, this catalyst effectively promotes the insertion of CO_2_ into the C−H bond of terminal alkynes under unprecedentedly mild conditions.^43^

**Scheme 26:**
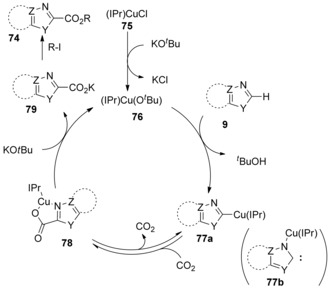
Proposed mechanism for (IPr)CuCl‐catalyzed C−H carboxylation.

Crucial intermediates **77 a** and **78** in Scheme 26 were isolated by Hou and co‐workers to elucidate the mechanism for (IPr)CuCl‐catalyzed C−H carboxylation.^41^ The authors showed that complex **78** could be obtained from **77 a** by exposing it to 1 atm of CO_2_ pressure at room temperature. A proposed catalytic cycle is presented in Scheme 26. Initially, ligand exchange between (IPr)CuCl (**75**) and KO*t*Bu affords (IPr)Cu(O*t*Bu) (**76**), which deprotonates the heterocycle to form **77 a**. CO_2_ insertion of **77 a** takes place to afford **78**, which subsequently reacts with KO*t*Bu to regenerate catalyst (IPr)Cu(O*t*Bu) (**76**), and produces the potassium carboxylate **79**. Ariafard, On the basis of DFT studies Yates and co‐workers suggested an alternative mechanism in which carbene intermediate **77 b** was generated in the catalytic cycle, instead of **77 a**.^44^ The authors indicated that **77 b** is more reactive toward CO_2_ insertion reaction through nucleophilic attack. Similarly to the Au‐catalyzed direct carboxylation, this method could also be regarded as a C−H deprotonation process.

**Scheme 27:**
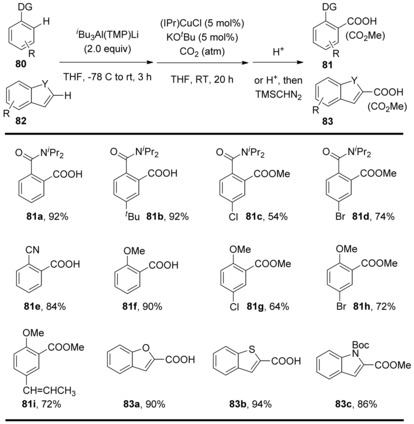
Selected examples of Cu‐catalyzed formal C−H carboxylation.

More recently, Hou and co‐workers developed a method for the C−H carboxylation of various aromatic compounds with CO_2_.^46^ The carboxylation products were afforded by deprotonative alumination using an *i*Bu_3_Al(TMP)Li aluminate base^45^ followed by the carboxylation of the resulting arylaluminum species with a standard atmospheric CO_2_ pressure in the presence of a catalytic amount of an NHC–copper complex and KO*t*Bu. A relatively broad scope of benzene derivatives, such as *N*,*N*‐diisopropylbenzamide, benzonitrile, and anisole, bearing both electron‐withdrawing and electron‐donating groups, undergo the alumination/carboxylation sequence to afford the corresponding carboxylic acids in high yield and high selectivity (Scheme 27, **81 a**–**i**). Heteroarenes such as benzofuran, benzothiophene, and indole derivatives are also compatible with this method (Scheme 27, **83 a**–**c**).

**Scheme 28:**
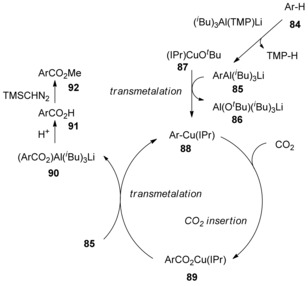
Proposed mechanism for Cu‐catalyzed formal C−H carboxylation.

To understand the mechanism of the reaction, some key intermediates such as the copper aryl and copper isobutyl complexes (and their carboxylation products) were isolated and structurally characterized by X‐ray crystallography. A plausible mechanism is shown in Scheme 28. Initially, the arylaluminum species **85** would be generated by the deprotonation of an aromatic compound **84** in the presence of aluminate reagent *i*Bu_3_Al(TMP)Li.^45^ The arylaluminum species **85** could then undergo transmetallation with (IPr)CuO*t*Bu (**87**; formed by treatment of IPrCuCl with KO*t*Bu) to give the copper aryl species **88** with release of Al(O*t*Bu)(*i*Bu)_3_Li (**86**). Under CO_2_ atmosphere, the carboxyl would be inserted into the Ar−Cu bond of the copper aryl species **88** to give copper carboxylate complex **89**. Lastly, the transmetallation between the complex **89** and another molecule of the arylaluminum species **85** would give the aluminum carboxylate species **90**, simultaneously regenerating arylcopper species **88**. The carboxylic acid **91** would be obtained by the hydrolysis of aluminum carboxylate species **90**, and the ester derivative ArCOOMe (**92**) would be achieved by treatment of carboxylic acid **91** with trimethylsilyldiazomethane (TMSCHN_2_). This reaction would be classified by the C−H deprotonation‐type mechanism.

#### 1.3.3 Rh‐catalyzed C−H carboxylation

**Scheme 29:**
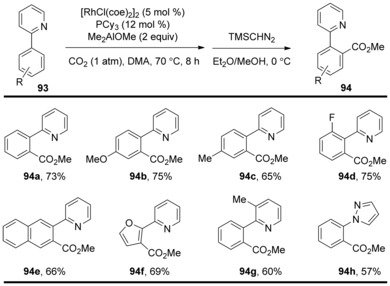
Selected examples of Rh‐catalyzed C−H carboxylation.

Besides gold and copper catalyzed C−H carboxylation, the research group of Iwasawa developed a method for Rh^I^‐catalyzed direct C−H carboxylation of aromatic compounds under ambient CO_2_ pressure through chelation‐assisted C−H activation.^47^ This reaction showed no limitation concerning the acidity of the C−H bond, as substrates bearing both electron‐donating and electron‐withdrawing groups are tolerated (Scheme 29). Pyridyl and pyrazolyl were used and found to also be suitable as directing groups, leading to the *ortho*‐carboxylated products in good yields. The use of a stoichiometric methylaluminum alkoxide AlMe_2_(OMe), prepared upon the mixing of Me_3_Al and MeOH, is important for efficient carboxylation. It is noteworthy that the formation of dicarboxylated product was not observed using this method.

**Scheme 30:**
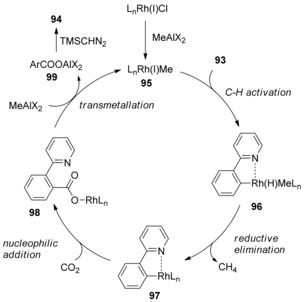
Proposed mechanism for Rh‐catalyzed C−H carboxylation.

A reasonable catalytic cycle proposed oxidative addition of Rh^I^ species **95** into the C−H bond of 2‐arylpyridine **93** to form Rh^III^ species **96,** followed by the reductive elimination of methane to give Rh^I^ species **97** (Scheme 30). Subsequently, nucleophilic carboxylation of arylrhodium **97** produces rhodium carboxylate **98**, which then undergoes transmetallation with methylaluminum to afford aluminum carboxylate **99**, regenerating methylrhodium catalyst **95**. Further reaction of aluminum carboxylate **99** with TMSCHN_2_ in methanol affords ester product **94**. Unlike the previously presented transition‐metal‐catalyzed processes, this proposed mechanism would fall under the C−H oxidative‐addition‐type pathway.

More recently, Iwasawa and co‐workers reported a method for Rh‐catalyzed C−H carboxylation of simple arenes under atmospheric CO_2_ pressure without the assistance of a directing group.^48^ The authors found that the bidentate ligand was essential for this reaction, since bulky monodentate phosphine complexes showed no reactivity. The use of AlMe_1.5_(OEt)_1.5_, which was prepared by mixing AlMe_3_ and EtOH, dramatically improved the efficiency of the reaction. Adding 1,1,3,3‐tetramethylurea (TMU) can also promote the carboxylation, probably owing to the stabilization of coordinatively unsaturated Rh^I^ species. It is important to note that polar solvents play important roles, since the reaction resulted in poor turnover number (TON) in the absence of dimethylacetamide (DMA). This method allows various arenes to undergo directed carboxylation to afford the corresponding carboxylic acids with moderate TON. Impressively, even simple benzoic acid could be obtained by treatment of benzene under the Rh‐catalyzed conditions (Table 3; **91 a**). Electron‐donating groups such as methyl or methoxy led to mixed products whereby the *meta*‐carboxylated product is the major product (Table 3; **91 b** and **91 c**). Electron‐withdrawing‐group‐substituted arenes such as halobenzenes mainly afforded *ortho*‐carboxylated products (Table 3; **91 d**). Furthermore, benzofuran, *N*‐Boc indole, and ferrocene were also found to be compatible, giving the corresponding carboxylic acids in acceptable yields (Table 3, **91 e**–**g**).

**Table 3:** Rh‐catalyzed C−H carboxylation of simple arenes. 
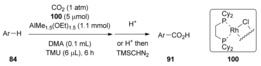

Reactant	*T* [°C]	Main product	TON [%]	Regioselectiviy [α:β:γ]
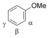		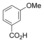		
**84 a**	85	**91 a**	37	–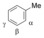		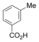		
**84 b**	120	**91 b**	18	17:57:26
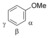		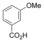		
**84 c**	145	**91 c**	39	23:54:23
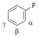		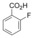		
**84 d**	85	**91 d**	39	59:29:12
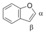		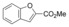		
**84 e**	145	**91 e**	12	100:0:0
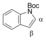		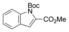		
**84 f**	145	**91 f**	21	86:14:0
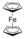		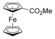		
**84 g**	145	**91 g**	60	–

**Scheme 31:**
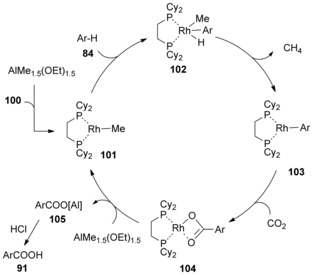
Proposed mechanism for Rh‐catalyzed carboxylation of simple arenes.

Competitive reactions of benzene and deuterated benzene were carried out by Iwasawa et al. finding a kinetic isotope effect (KIE) value of 5.5 after 1 h reaction time. This result suggests that the rate‐determining step is C−H bond activation. On the basis of these results and literature precedent,6b, 49 a tentatively reaction mechanism was proposed by the Iwasawa et al. (Scheme 31). Initially, the methylrhodium(I) complex **101** was generated by the mixture of Rh catalyst **100** and AlMe_1.5_(OEt)_1.5_. Oxidative addition of a C−H bond of the arene affords an aryl(hydride)(methyl)rhodium(III) intermediate (**102**). Reductive elimination of intermediate **102** provides arylrhodium(I) complex **103** and methane, which was detected by GC analysis. Nucleophilic addition of the highly reactive arylrhodium(I) complex **103** to CO_2_ gives rhodium(I) benzoate complex **104**, which then further reacts with AlMe_1.5_(OEt)_1.5_ to furnish the aluminum carboxylate **105** and regenerating the active Rh catalyst **101**. The final product would be obtained by simple acidic workup.

Another detailed mechanistic study by Iwasawa and co‐workers confirmed their initially postulated mechanism above to be fundamentally correct.^50^ However, instead of the reaction between **104** and methylaluminum species to give **101**, the transmetallation of **104** with chloroaluminum species could also occur, converting the catalyst back to **100**. The carboxylation of catalyst **101** could also take place in the same manner as the carboxylation of **103**, consequently affording the acetic acid by‐product.

### 1.4 Enzymatic carboxylation

Biocatalysis holds enormous potential as a useful supplementary technology for the chemical industry. It simplifies the manufacturing processes for some reactions that are not easily conducted by classical organic synthesis. The highly reactive and selective bioconversions can also lead to more economical and more environmentally friendly processes.^51^

**Scheme 32:**
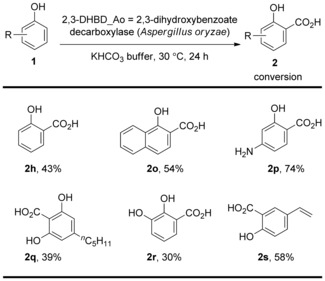
Selected examples of (de)carboxylase‐catalyzed carboxylation of phenols.

In 2012, the research group of Faber developed a method for direct C−H carboxylation of phenols catalyzed by (de)carboxylases in bicarbonate media under extremely mild conditions.^52^ 2,3‐Dihydroxybenzoate decarboxylase (2,3‐DHBD_Ao; *Aspergillus oryzae*)^53^ catalyzed the carboxylation shown in Scheme 32. This method allows phenols bearing alkane, hydroxy, and vinyl groups to undergo selective *ortho*‐carboxylation to give the corresponding salicylic acids in moderate conversions. 2,6‐Dihydroxybenzoate decarboxylase (2,6‐DHBD_Rs; *Rhizobium sp*.)^54^ and salicylic acid decarboxylase (SAD Tm; *Trichosporon moniliiforme*)^55^ were also found to be efficient for the carboxylation process. The phenolic acid decarboxylase derived from *Lactobacillus plantarum* (PAD_Lp)^56^ and *Bacillus amyloliquefaciens* (PAD_Ba)^57^ selectively acted at the β‐carbon atom of styrenes forming (*E*)‐cinnamic acids. Overall, this method for enzymatic carboxylation of phenols represents a promising biocatalytic equivalent to the classical Kolbe–Schmitt reaction to prepare the corresponding salicylic acid derivatives at low temperature and ambient pressure.

**Scheme 33:**
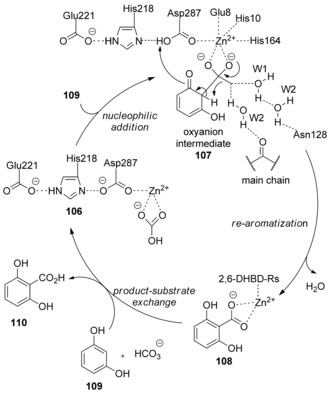
Proposed mechanism for decarboxylase‐catalyzed *ortho*‐carboxylation of phenols.

On the basis of the crystal structure of 2,6‐dihydroxybenzoate decarboxylase (Strain MTP‐1005; PDB code 2DVU) reported by Goto and co‐workers,^58^ it was suggested that proton abstraction of the phenolic hydroxy group occurs at the Asp287 residue, activated in a triad by His218 and Glu221 (Scheme 33). This in turn enhances the nucleophilicity of the *ortho* carbon and enables a nucleophilic attack onto coordinated bicarbonate bound by a tightly coordinated Zn^2+^ center bound by Asp287, Glu8, His10, and His164 residues. The oxyanion intermediate **107** is formed initially through nucleophilic addition of 1,5‐dihydroxybenzene (**109**) to the active site of 2,6‐dihydroxybenzoate decarboxylase (**106**). The carboxylated product 2,6‐dihydroxybenzoic acid docked into the **108** active site is then obtained by the re‐aromatization of **107** aided by a network of three catalytically important and structurally conserved water molecules (W1, W2, and W3), which is triggered by Asn128 through a protonation–deprotonation sequence. The final carboxylated product **110** would be released from the active site by replacing another substrate **109** with **108**, simultaneously regenerating the decarboxylase with an undocked active site for a subsequent catalytic cycle. This enzymatic carboxylation relies on the electrophilic aromatic substitution process.

## 2 Conclusions

The direct carboxylation of hydrocarbons by way of fixing CO_2_ as a C1 source holds potential in organic synthesis as an ideal strategy to construct the C−C bond in a step‐ and atomeconomical fashion. Recent advances in C−H carboxylation using CO_2_ were summarized; in particular the carboxylation of aromatic compounds that are important motifs among natural products and biologically active compounds. This progress represents the development of green chemistry methodologies to meet economic and environmental requirements, and provides an alternative to traditional CO_2_ coupling reactions that require organometallic reagents.

The classic strategies for C−H carboxylation with CO_2_ require the use of base or Lewis‐acid mediators. In this regard, the base‐mediated C−H carboxylation usually requires a strong base to deprotonate the most acidic proton to form a strong nucleophilic carbon atom, thus enabling attack on the weakly electrophilic CO_2_. However, this base‐mediated C−H carboxylation generally requires relatively high reaction temperatures. On the other hand the strategy of Lewis‐acid‐mediated C−H carboxylation relies on the activation of CO_2_ through coordination with the Lewis acid. The aromatic compounds can therefore react with the activated CO_2_. This strategy allows for C−H carboxylation at relatively low reaction temperatures. However, it usually requires high CO_2_ pressure and the regioselectivity is often poor.

The development of methods operating under milder conditions, such as room temperature or low pressure of CO_2_, still remains a great challenge for synthetic chemists. Many catalytic transformations of CO_2_ have been accomplished in recent decades, and this progress allows for C−H carboxylation to proceed at relatively low reaction temperatures and CO_2_ pressures. Despite advances in this area the substrate scope is often limited. Thus, it is still highly desirable to develop new methodologies for carboxylation to proceed in simple, mild, and generally applicable conditions using a broad scope of readily available starting materials.

## Conflict of interest

*The authors declare no conflict of interest*.

## Biographical Information

Junfei Luo received his B.A degree in Pharmacy from East China University Of Science and Technology, P.R. China in 2010 under the guidance of Prof. Weiping Deng, and received his Ph.D degree in chemistry from Queen Mary University of London, United Kingdom in 2015 under the supervision of Prof. Igor Larrosa. His graduate research focused on C−H carboxylation with CO_2_ and carboxyl‐group‐directed C−H activation as a strategy for the synthesis of *meta*‐substituted phenol derivatives. In July 2016, he started his current position as an A/Prof. at Ningbo University, P.R. China.

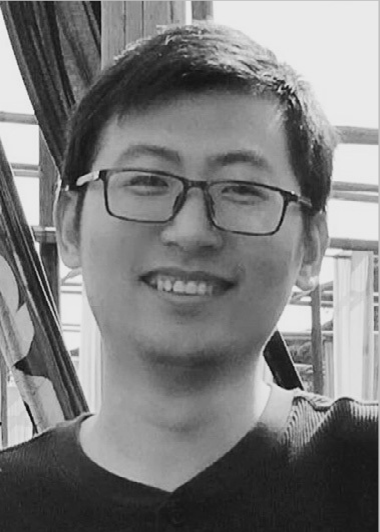

## Biographical Information

Igor Larrosa graduated in Chemistry from the University of Barcelona in 1999, where he also completed MChem (2000) and Ph.D. degrees (2004) with Prof. Felix Urpi and Prof. Pere Romea. After a research period in Prof. Erick M. Carreira′s laboratories at ETH (Zurich), Igor moved to Imperial College London as a Postdoctoral Researcher to work in Prof. Anthony G. M. Barrett′s group. In September 2007 he started his independent career as a Lecturer at Queen Mary University of London. In 2011, Igor was awarded a European Research Council starting grant. Since 2014 Igor holds a Chair in Organic Chemistry at the University of Manchester.